# Deep Learning to Assess Long-term Mortality From Chest Radiographs

**DOI:** 10.1001/jamanetworkopen.2019.7416

**Published:** 2019-07-19

**Authors:** Michael T. Lu, Alexander Ivanov, Thomas Mayrhofer, Ahmed Hosny, Hugo J. W. L. Aerts, Udo Hoffmann

**Affiliations:** 1 Cardiovascular Imaging Research Center, Department of Radiology, Massachusetts General Hospital, Harvard Medical School, Boston; 2 School of Business Studies, Stralsund University of Applied Sciences, Stralsund, Germany; 3 Department of Radiation Oncology and Radiology, Dana Farber Cancer Institute, Brigham and Women’s Hospital, Harvard Medical School, Boston, Massachusetts

## Abstract

**Importance:**

Chest radiography is the most common diagnostic imaging test in medicine and may also provide information about longevity and prognosis.

**Objective:**

To develop and test a convolutional neural network (CNN) (named CXR-risk) to predict long-term mortality, including noncancer death, from chest radiographs.

**Design, Setting, and Participants:**

In this prognostic study, CXR-risk CNN development (n = 41 856) and testing (n = 10 464) used data from the screening radiography arm of the Prostate, Lung, Colorectal, and Ovarian Cancer Screening Trial (PLCO) (n = 52 320), a community cohort of asymptomatic nonsmokers and smokers (aged 55-74 years) enrolled at 10 US sites from November 8, 1993, through July 2, 2001. External testing used data from the screening radiography arm of the National Lung Screening Trial (NLST) (n = 5493), a community cohort of heavy smokers (aged 55-74 years) enrolled at 21 US sites from August 2002, through April 2004. Data analysis was performed from January 1, 2018, to May 23, 2019.

**Exposure:**

Deep learning CXR-risk score (very low, low, moderate, high, and very high) based on CNN analysis of the enrollment radiograph.

**Main Outcomes and Measures:**

All-cause mortality. Prognostic value was assessed in the context of radiologists’ diagnostic findings (eg, lung nodule) and standard risk factors (eg, age, sex, and diabetes) and for cause-specific mortality.

**Results:**

Among 10 464 PLCO participants (mean [SD] age, 62.4 [5.4] years; 5405 men [51.6%]; median follow-up, 12.2 years [interquartile range, 10.5-12.9 years]) and 5493 NLST test participants (mean [SD] age, 61.7 [5.0] years; 3037 men [55.3%]; median follow-up, 6.3 years [interquartile range, 6.0-6.7 years]), there was a graded association between CXR-risk score and mortality. The very high-risk group had mortality of 53.0% (PLCO) and 33.9% (NLST), which was higher compared with the very low-risk group (PLCO: unadjusted hazard ratio [HR], 18.3 [95% CI, 14.5-23.2]; NLST: unadjusted HR, 15.2 [95% CI, 9.2-25.3]; both *P* < .001). This association was robust to adjustment for radiologists’ findings and risk factors (PLCO: adjusted HR [aHR], 4.8 [95% CI, 3.6-6.4]; NLST: aHR, 7.0 [95% CI, 4.0-12.1]; both *P* < .001). Comparable results were seen for lung cancer death (PLCO: aHR, 11.1 [95% CI, 4.4-27.8]; NLST: aHR, 8.4 [95% CI, 2.5-28.0]; both *P* ≤ .001) and for noncancer cardiovascular death (PLCO: aHR, 3.6 [95% CI, 2.1-6.2]; NLST: aHR, 47.8 [95% CI, 6.1-374.9]; both *P* < .001) and respiratory death (PLCO: aHR, 27.5 [95% CI, 7.7-97.8]; NLST: aHR, 31.9 [95% CI, 3.9-263.5]; both *P* ≤ .001).

**Conclusions and Relevance:**

In this study, the deep learning CXR-risk score stratified the risk of long-term mortality based on a single chest radiograph. Individuals at high risk of mortality may benefit from prevention, screening, and lifestyle interventions.

## Key Points

**Question:**

Is a convolutional neural network able to extract prognostic information from chest radiographs?

**Findings:**

In this prognostic study of data from 2 randomized clinical trials (Prostate, Lung, Colorectal, and Ovarian Cancer Screening Trial [n = 10 464] and National Lung Screening Trial [n = 5493]), a convolutional neural network identified persons at high risk of long-term mortality based on their chest radiographs, even with adjustment for the radiologists' diagnostic findings and standard risk factors.

**Meaning:**

Individuals at high risk of mortality based on chest radiography may benefit from prevention, screening, and lifestyle interventions.

## Teaser

This prognostic study develops and tests a convoluted neural network (CXR-risk) to predict long-term mortality from chest radiographs.

## Introduction

Chest radiography is the most common diagnostic imaging test in medicine.^1^ Chest radiography is especially common in older adults; in 2013, there were 1039 outpatient chest radiographs per 1000 US Medicare Part B beneficiaries.^2^ Most chest radiographs are reported as normal, in that they rule out a specific diagnosis such as pneumonia. However, even normal radiographs manifest additional minor abnormalities, such as aortic calcification^3^ or an enlarged heart,^4,5^ that may provide a new window into prognosis and longevity^6^ with the potential to inform decisions about lifestyle, screening, and prevention.^7^ Whereas physicians may interpret thousands of chest radiographs during a career, they rarely know the outcomes in these patients a decade later. Therefore, it is difficult to develop an intuition to articulate which features have long-term prognostic value.

The traditional approach to identify prognostic imaging biomarkers has been to hypothesize that an individual finding has value, manually assess the finding, and test its association with the outcome. Deep learning, a type of artificial intelligence in which data are fed through many layers with the composition of each layer learned automatically from large data sets, allows for a new approach that evaluates the entire image without human guidance to differentiate what findings have value.^8,9^ Deep learning models have been developed to make diagnoses based on chest radiography, such as pneumonia, with the radiologists’ findings as the reference standard.^10,11,12,13,14,15,16^ However, whether deep learning can reach beyond diagnosis to assess long-term prognosis from chest radiographs is not known.

To test the hypothesis that a deep learning model can extract prognostic information from diagnostic radiographs, we developed a convolutional neural network (CNN) named CXR-risk to predict 12-year mortality from chest radiographs. The final model was tested in 2 well-established, multicenter clinical trials of screening chest radiography: the Prostate, Lung, Colorectal, and Ovarian Cancer Screening Trial (PLCO)^17^ and the National Lung Screening Trial (NLST).^18^

## Methods

### Trial Data Sets

In this prognostic study, the CXR-risk CNN was developed and tested using data from the screening radiography arm of the PLCO trial (n = 52 320), a community cohort of asymptomatic nonsmokers and smokers (aged 55-74 years) enrolled at 10 US sites from November 8, 1993, through July 2, 2001.^17,19^ External testing used data from the screening radiography arm of the NLST (n = 5493), a community cohort of heavy smokers (aged 55-74 years) enrolled at 21 US sites from August 2002, through April 2004.^18^ Data analysis was performed from January 1, 2018, to May 23, 2019. The PLCO and NLST participants provided written informed consent for the original trials. Secondary use of PLCO and NLST data was approved by the National Cancer Institute, Bethesda, Maryland, and Partners Healthcare, Boston, Massachusetts institutional review board.^20^ Secondary use of chest radiographs from the NLST was further approved by the American College of Radiology Imaging Network (ACRIN). This study followed the Transparent Reporting of a Multivariable Prediction Model for Individual Prognosis or Diagnosis (TRIPOD) reporting guideline.

**Figure 1.**
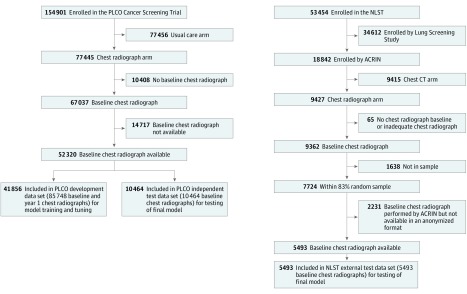
Data Sets for Deep Learning Model Development and Testing The Prostate, Lung, Colorectal, and Ovarian (PLCO) trial development data set includes all baseline and year 1 chest radiographs, with several participants having more than 1 chest radiograph from either time point. The PLCO and National Lung Screening Trial (NLST) testing data sets include a single baseline chest radiograph per person. ACRIN indicates American College of Radiology Imaging Network; CT, computed tomography.

The CXR-risk CNN development and the first round of testing (Figure 1) were performed in the screening chest radiograph arm of the PLCO trial.^17,19^ Major exclusion criteria included a history of prostate, lung, colorectal, or ovarian cancer or current treatment for any cancer (excluding basal and squamous cell skin cancer). Participants were randomized to annual chest radiography screening vs no screening; the trial’s primary finding was that screening chest radiography did not reduce lung cancer mortality.^17^ Participants had baseline (T0) and up to 3 yearly chest radiographs (T1-T3). Participants whose baseline chest radiographs were available from the National Cancer Institute (n = 52 320) were included. Of these patients, 41 856 (80%) were randomly assigned for model development (PLCO development data set); the remaining 10 464 patients (20%) were reserved for testing of the final model (PLCO test data set).

The final model was further externally tested in the chest radiograph arm of NLST (Figure 1).^18^ In contrast with PLCO, which included nonsmokers and smokers, NLST enrolled only current and recent (smoking cessation within the past 15 years) former heavy smokers with a 30 pack-year or more smoking history. Major exclusion criteria included a history of lung cancer or treatment for any cancer (excluding nonmelanoma skin cancer or carcinoma in situ) within the past 5 years.^18,21^ Participants were randomized to screening chest radiography vs low-dose chest computed tomography; the trial’s primary finding was that chest computed tomography reduced lung cancer mortality by 20% compared with chest radiography.^18^ Similar to PLCO, baseline (T0) and yearly (T1-T2) chest radiographs were obtained. We included an 83% random sample from 21 sites whose baseline chest radiographs were available (NLST test data set [n = 5493]) from ACRIN.

### Standard Risk Factors and Diagnostic Chest Radiograph Findings

Baseline risk factors, including age, sex, smoking status, diabetes, hypertension, obesity (body mass index [BMI] ≥30 [calculated as weight in kilograms divided by height in meters squared]), underweight (BMI <18.5), and previous myocardial infarction, stroke, or cancer, were self-reported. Upright posterior-anterior chest radiographs were interpreted locally by centrally qualified radiologists for potentially significant diagnostic findings, including lung nodules, major atelectasis, pleural plaque or effusion, lymphadenopathy, chest wall or bony lesion, chronic obstructive pulmonary disease or emphysema, lung opacity, cardiomegaly or other cardiovascular abnormality, and lung fibrosis. The radiologists’ findings were provided to the participants and their physicians.^18,19^

### Outcomes

The primary outcome was all-cause mortality. Participants were followed up until December 31, 2009, or for up to 13 years (PLCO) or 8 years (NLST).^17,18^ Death and incident cancer were assessed via annual questionnaire, supplemented by communication with next of kin and linkage to the National Death Index. The secondary outcome was cause-specific mortality, as reported in the parent trials (eMethods in the Supplement).^18,22^

### Data Sets for CNN Development and Testing

The CXR-risk CNN was developed in an 80% (41 856 of 52 320) random sample from PLCO participants with a baseline chest radiograph (Figure 1). Development data set participants were further randomly divided for model training (33 485 of 41 856 [80%]) and tuning (8371 [20%]). Each development data set participant’s baseline and T1 chest radiographs were treated independently (n = 85 748), with some participants having more than 1 baseline or T1 chest radiograph. The final model was tested in the remaining 20% (10 464 of 52 320) of PLCO participants held out during model development as an independent test data set (PLCO test).^23^ The model was further externally tested in 5493 NLST participants (NLST test). Both test data sets included a single baseline chest radiograph per participant to reflect the anticipated use case.

### CNN Development

We used a transfer learning approach with a modified Inception-v4 architecture.^24^ Image preprocessing, staged classifier, training hyperparameters, and implementation of the model are described in the eMethods in the Supplement. The CNN was developed using the chest radiographs and the staged classifier only; no other information, including age, sex, risk factors, chest radiograph findings, duration of follow-up, or censoring, was available to the CNN. Gradient-weighted class activation maps (Grad-CAM) were generated to localize the anatomy that contributed to predictions.^25^

### The CXR-Risk Score

The CXR-risk CNN takes as input a single chest radiograph image; the output is a continuous CXR-risk probability (probability of death between 0 and 1). To facilitate interpretability of the survival analysis, this output was converted to an ordinal CXR-risk score based on quantile thresholds set in the PLCO development data set and then applied to the PLCO and NLST test data sets (eTable 1 in the Supplement). The bottom first, second, and third quartiles corresponded to the very low-, low-, and moderate-risk categories. The top 75th through 95th percentile was assigned as high risk, and the top 95th and above percentile was considered as very high risk.

### Test-Retest Reliability on Repeated Chest Radiographs

During the quality control process, several participants’ chest radiographs were repeated, usually because the original did not include the entire lung or was overexposed. These images allowed an analysis of test-retest reliability. The PLCO test participants who had multiple T1 chest radiographs were chosen because these chest radiographs were not used in model development or testing. The chest radiographs were manually reviewed to exclude duplicates.

### Statistical Analysis

We determined the association between the CXR-risk score and all-cause mortality (primary outcome) using Cox proportional hazards regression models and Kaplan-Meier curves. We estimated hazard ratios (HRs) and 95% CIs, both unadjusted and then adjusted for 9 diagnostic chest radiograph findings (noncalcified lung nodule, major atelectasis, pleural plaque or effusion, lymphadenopathy, chest wall or bony lesion, lung opacity, emphysema or chronic obstructive pulmonary disease, cardiomegaly or other cardiovascular abnormality, and lung fibrosis) and 10 standard risk factors (age, sex, smoking category [current, former, or never], diabetes, hypertension, obesity, underweight, and previous myocardial infarction, stroke, or cancer). Risk factors and findings were prospectively selected as those available in both trials with likely prognostic value. Subgroup analyses included those healthy or unhealthy at baseline (defined as previous myocardial infarction, stroke, or cancer at enrollment) and in 5-year age and sex strata. Cox proportional hazards regression models were constructed for secondary outcomes of cause-specific mortality due to lung cancer, nonlung cancer, cardiovascular illness, and respiratory illness. The proportional hazards assumption was tested with Schoenfeld residuals.^26^ Goodness of fit was assessed using the test by Grønnesby and Borgan^27^ without gross model violations.

To assess discrimination for all-cause mortality, nested area under the receiver operating characteristic curves (AUCs) with and without the continuous CXR-risk were compared using the method by DeLong et al.^28^ The continuous net reclassification improvement of adding CXR-risk to radiograph findings, risk factors, and findings plus risk factors was calculated using the risk prediction (incrisk)^29^ package. Bootstrap standard errors and 95% CIs were calculated using 1000 bootstrap samples.^30^ Calibration was assessed by plotting mean predicted vs observed mortality within deciles of CXR-risk.^31^ For PLCO, 12-year predicted mortality was compared with 12-year observed mortality. For NLST, 12-year predicted mortality was compared with 6-year observed mortality.

Interradiograph test-retest reliability was estimated with the intraclass correlation coefficient of the continuous CXR-risk probability computed using a 2-way mixed-effects model with absolute agreement for an individual measurement. The primary outcome was the HR for all-cause mortality, with a threshold of significance of *P* < .05. *P* values were 2-sided. Statistical analysis was performed with Stata, version 14.2 (StataCorp).

## Results

### Baseline Risk Factors and Chest Radiographs

Of 10 464 PLCO trial data set participants, 5405 (51.6%) were men with a mean (SD) age of 62.4 (5.4) years. Of 5493 NLST test data set participants, 3037 (55.3%) were men, with a mean (SD) age of 61.7 (5.0) years. Baseline risk factors and radiograph findings for the PLCO development, PLCO test, and NLST test data sets are presented in Table 1. Subsequent results are reported for PLCO test and NLST test data sets only.

**Table 1.** Baseline Risk Factors, Radiographic Findings, and Outcomes^a^

Characteristic	PLCO		NLST
	Development (Training and Tuning) (n = 41 856)	Independent Test (n = 10 464)	External Test (n = 5493)
Chest radiographs, No.^b^	85 748	10 464	5493
Age, mean (SD), y	62.4 (5.4)	62.4 (5.4)	61.7 (5.0)
Male	21 648/41 856 (51.7)	5404/10 464 (51.6)	3037/5493 (55.3)
Race/ethnicity			
White, non-Hispanic	36 295 (86.7)	9049 (86.5)	5105 (92.9)
Black, non-Hispanic	2451 (5.9)	642 (6.1)	221 (4.0)
Hispanic	775 (1.9)	207 (2.0)	49 (0.9)
Asian	1895 (4.5)	452 (4.3)	39 (0.7)
Other or unknown	440 (1.1)	114 (1.1)	79 (1.4)
Smoking			
Never	18 598/41 776 (44.5)	4724/10 445 (45.2)	NA
Former	18 750/41 776 (44.9)	4580/10 445 (43.9)	2769/5493 (50.4)
Current	4428/41 776 (10.6)	1141/10 445 (10.9)	2724/5493 (49.6)
Diabetes	3217/41 635 (7.7)	749/10 413 (7.2)	505/5481 (9.2)
Hypertension	13 937/41 635 (33.5)	3445/10 418 (33.1)	2021/5478 (36.9)
Obesity, BMI ≥30	9978/41 275 (24.2)	2513/10 326 (24.3)	1518/5484 (27.7)
Underweight, BMI <18.5	281/41 275 (0.68)	76/10 326 (0.74)	45/5484 (0.82)
Previous event			
Myocardial infarction^c^	3609/41 625 (8.7)	924/10 410 (8.9)	676/5470 (12.4)
Stroke	922/41 638 (2.2)	252/10 414 (2.4)	176/5470 (3.2)
Cancer	1824/41 779 (4.4)	431/10 445 (4.1)	228/5448 (4.2)
Baseline chest radiograph findings			
Lung nodule	3080/41 851 (7.4)	813/10 461 (7.8)	518/5493 (9.4)
Granuloma or benign calcified nodule	4508/41 851 (10.8)	1102/10 461 (10.5)	660/5493 (12.0)
Major atelectasis	19/41 851 (0.1)	6/10 461 (0.1)	16/5493 (0.3)
Pleural plaque or effusion	1464/41 851 (3.5)	385/10 461 (3.7)	266/5493 (4.8)
Lymphadenopathy	234/41 851 (0.6)	59/10 461 (0.6)	16/5493 (0.3)
Chest wall or bony abnormality	1831/14 851 (4.4)	433/10 461 (4.1)	22/5493 (0.4)
Lung opacity	320/41 851 (0.8)	76/10 461 (0.7)	9/5493 (0.2)
Emphysema or COPD	1084/41 851 (2.6)	257/10 461 (2.5)	810/5493 (14.8)
Cardiomegaly or other cardiovascular abnormality	1637/41 851 (3.9)	391/10 461 (3.7)	62/5493 (1.1)
Lung fibrosis	3124/41 851 (7.5)	810/10 461 (7.7)	372/5493 (6.8)
Other	4284/4851 (10.2)	1118/10 461 (10.7)	733/5493 (13.3)
Outcomes			
Follow-up, median (IQR), y	12.2 (10.5-12.9)	12.2 (10.5-12.9)	6.3 (6.0-6.7)
Mortality	5416/41 856 (12.9)	1402/10 464 (13.4)	374/5493 (6.8)

Abbreviations: BMI, body mass index (calculated as weight in kilograms divided by height in meters squared); COPD, chronic obstructive pulmonary disease; IQR, interquartile range; NA, not applicable; NLST, National Lung Screening Trial; PLCO, Prostate, Lung, Colorectal, and Ovarian Trial.

^a^ Data are presented as No./total No. (%) of patients unless otherwise indicated.

^b^ The PLCO development data set includes all available baseline and year 1 chest radiographs. The PLCO test and NLST test data sets include the baseline chest radiographs only.

^c^ In the NLST data set, this field includes both previous myocardial infarction and heart disease.

### Vital Status

Median follow-up in the PLCO test data set was 12.2 years (interquartile range [IQR], 10.5-12.9 years). The all-cause mortality rate was 13.4% (1402 of 10 464 persons) for 117 619 person-years of follow-up. The NLST had half the median follow-up (6.3 years [IQR, 6.0-6.7 years]) and mortality (6.8% [374 of 5493 persons]) for 33 695 person-years. The number of deaths per 1000 person-years (Table 2) was similar in the PLCO data set (11.9 deaths; 95% CI, 11.3-12.6 deaths) and NLST data set (11.1 deaths; 95% CI, 10.0-12.3 deaths).

**Table 2.** Mortality Based on CXR-Risk Score

CXR-Risk Score	Mortality, No./Total No. (%)	Deaths per 1000 Person-Years (95% CI)	Unadjusted		Adjusted	
			HR (95% CI)	*P* Value	HR (95% CI)^a^	*P* Value
**PLCO Test Data Set (12-y Follow-up)**						
Very low	97/2543 (3.8)	3.3 (2.7-4.1)	1 [Reference]	NA	1 [Reference]	NA
Low	216/2769 (7.8)	6.8 (5.9-7.7)	2.0 (1.6-2.6)	<.001	1.4 (1.1-1.8)	.003
Moderate	339/2674 (12.7)	11.1 (10.0-12.4)	3.3 (2.7-4.2)	<.001	1.7 (1.3-2.2)	<.001
High	500/2006 (24.9)	23.0 (21.1-25.1)	7.0 (5.6-8.6)	<.001	2.6 (2.1-3.4)	<.001
Very high	250/472 (53.0)	57.4 (50.8-65.0)	18.3 (14.5-23.2)	<.001	4.8 (3.6-6.4)	<.001
Total	1402/10 464 (13.4)	11.9 (11.3-12.6)	NA	NA	NA	NA
**NLST Test Data Set (6-y Follow-up)**						
Very low	20/752 (2.7)	4.2 (2.7-6.6)	1 [Reference]	NA	1 [Reference]	NA
Low	64/1679 (3.8)	6.1 (4.8-7.8)	1.4 (0.9-2.4)	.16	1.2 (0.7-1.9)	.56
Moderate	115/1723 (6.7)	10.9 (9.1-13.1)	2.6 (1.6-4.1)	<.001	1.7 (1.0-2.8)	.03
High	114/1159 (9.8)	16.4 (13.6-20.0)	3.9 (2.4-6.3)	<.001	2.3 (1.4-3.7)	.002
Very high	61/180 (33.9)	62.8 (48.8-80.7)	15.2 (9.2-25.3)	<.001	7.0 (4.0-12.1)	<.001
Total	374/5493 (6.8)	11.1 (10.0-12.3)	NA	NA	NA	NA

Abbreviation: HR, hazard ratio; NA, not applicable; NLST, National Lung Screening Trial; PLCO, Prostate, Lung, Colorectal, and Ovarian Cancer Screening Trial.

^a^ Hazard ratios are adjusted for 9 chest radiograph findings (lung nodule, major atelectasis, pleural plaque or effusion, lymphadenopathy, chest wall or bony lesion, chronic obstructive pulmonary disease or emphysema, lung opacity, cardiomegaly or other cardiovascular abnormality, and lung fibrosis) and 10 risk factors (age, sex, smoking category, diabetes, hypertension, obesity, underweight, and previous myocardial infarction, stroke, and cancer).

### CXR-Risk Score and All-Cause Mortality

The CXR-risk score had a graded association with mortality (Table 2). In the PLCO data set, mortality rates were 3.8% (97 of 2543) in the very low-risk group, 7.8% (216 of 2769) in the low-risk group, 12.7% (339 of 2674) in the moderate-risk group, 24.9% (500 of 2006) in the high-risk group, and 53.0% (250 of 472) in the very high-risk group. In NLST, mortality rates were similar after accounting for the shorter duration of follow-up (very low-risk group: 2.7% [20 of 752]; low-risk group: 3.8% [64 of 1679]; moderate-risk group: 6.7% [115 of 1723]; high-risk group: 9.8% [114 of 1159]; very high-risk group: 33.9% [61 of 180]). Similar numbers of deaths per 1000 person-years in each CXR-risk category (Table 2) were noted: very low-risk group (3.3 [95% CI, 2.7-4.1] in the PLCO data set and 4.2 [95% CI, 2.7-6.6] in the NLST data set) and the very high-risk group (57.4 [95% CI, 50.8-65.0] in the PLCO data set and 62.8 [95% CI, 48.8-80.7] in the NLST data set).

**Figure 2.**
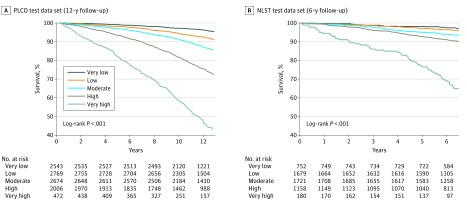
Kaplan-Meier Survival Estimates by CXR-Risk Score in the Prostate, Lung, Colorectal, and Ovarian Cancer Screening Trial (PLCO) and National Lung Screening Trial (NLST) Test Data Sets

Kaplan-Meier survival estimates based on the CXR-risk score are provided in Figure 2. We estimated HRs with 95% CIs for each CXR-risk category, with very low risk as the reference (Table 2). There was a graded increase in mortality with increasing CXR-risk score. Persons in the very high-risk group had higher mortality compared with those in the very low-risk group (PLCO data set: unadjusted HR, 18.3 [95% CI, 14.5-23.2]; NLST data set: unadjusted HR, 15.2 [95% CI, 9.2-25.3]; both *P* < .001). There was less unadjusted hazard associated with diabetes (PLCO data set: unadjusted HR, 2.7 [95% CI, 2.3-3.1]; *P* < .001; NLST data set: unadjusted HR, 1.9 [95% CI, 1.4-2.5]; *P* < .001), and finding a lung nodule on the chest radiograph (PLCO data set: unadjusted HR, 1.5 [95% CI, 1.3-1.8]; *P* < .001; NLST data set: unadjusted HR, 1.9 [95% CI, 1.5-2.5]; *P* < .001).

The association between CXR-risk score and death was robust to adjustment for the radiologists’ diagnostic findings (eg, lung nodule) and standard risk factors (eg, age, sex, and diabetes), as detailed in Table 2 and eTable 2 in the Supplement. In the very high-risk group, adjusted HRs (aHRs) were 4.8 (95% CI, 3.6-6.4; *P* < .001) in the PLCO data set and 7.0 (95% CI, 4.0-12.1; *P* < .001) in the NLST data set. The aHR associated with diabetes was smaller (PLCO: aHR, 1.7 [95% CI, 1.5-2.0]; *P* < .001; NLST data set: aHR, 1.5 [95% CI, 1.1-2.0]; *P* = .016), as was the aHR associated with lung nodule findings (PLCO data set: aHR, 1.3 [95% CI, 1.1-1.5]; *P* = .006; NLST data set: aHR, 1.6 [95% CI, 1.2-2.1]; *P* = .001) (eTable 3 in the Supplement).

Similar results were seen in stratified analyses of participants considered to be healthy at baseline (no previous myocardial infarction, stroke, or cancer). Among 8915 PLCO participants who were healthy at baseline, aHRs were 1.5 (95% CI, 1.1-1.9; *P* = .004) in the low-risk group, 1.7 (95% CI, 1.3-2.2; *P* < .001) in the moderate-risk group, 2.6 (95% CI, 2.0-3.4; *P* < .001) in the high-risk group, and 4.8 (95% CI, 3.5-6.6; *P* < .001) in the very high-risk group. Among the 4427 NLST participants who were healthy at baseline, aHRs were 1.1 (95% CI, 0.6-1.8; *P* = .78) in the low-risk group, 1.4 (95% CI, 0.8-2.3; *P* = .25) in the moderate-risk group, 1.9 (95% CI, 1.1-3.3; *P* = .02) in the high-risk group, and 4.8 (95% CI, 2.6-8.9; *P* < .001) in the very high-risk group. The association between CXR-risk and death remained across age and sex strata (eFigure 1 in the Supplement).

### Cause-Specific Mortality

Cause-specific mortality is provided in eTable 4 in the Supplement. In the PLCO data set, the most common cause of death was cardiovascular illness (4.1% [432 of 10 464]); in the NLST data set, the most common cause of death was lung cancer (2.1% [113 of 5493]). In both PLCO and NLST data sets, after adjustment for risk factors and radiologists’ findings, patients in the very high-risk group were significantly more likely to die of lung cancer (PLCO data set: aHR, 11.1 [95% CI, 4.4-27.8]; NLST data set: aHR, 8.4 [95% CI, 2.5-28.0]; both *P* ≤ .001), cardiovascular illness (PLCO data set: aHR, 3.6 [95% CI, 2.1-6.2]; NLST data set: aHR, 47.8 [95% CI, 6.1-374.9]; both *P* < .001), and respiratory illness (PLCO data set: aHR, 27.5 [95% CI, 7.7-97.8]; *P* < .001; NLST data set: aHR, 31.9 [95% CI, 3.9-263.5]; *P* = .001).

### Discrimination, Reclassification, and Calibration

Discrimination for all-cause mortality was assessed with nested AUCs (eTable 5 in the Supplement). The CXR-risk AUC was 0.75 for 12-year mortality in the PLCO data set and 0.68 for 6-year mortality in the NLST data set. Addition of CXR-risk was associated with significant AUC improvements compared with chest radiograph findings (PLCO data set: 0.58 to 0.74; *P* < .001; NLST data set: 0.59 to 0.70; *P* < .001), risk factors (PLCO data set: 0.76 to 0.78; *P* < .001; NLST data set: 0.68 to 0.72; *P* < .001), and combined risk factors plus findings (PLCO data set: 0.76 to 0.78; *P* < .001; NLST data set: 0.70 to 0.73; *P* < .001). Corresponding continuous net reclassification improvements associated with adding CXR-risk to findings (PLCO data set: 0.59; NLST data set: 0.44), risk factors (PLCO data set: 0.21; NLST data set: 0.32), and combined risk factors plus findings (PLCO data set: 0.20; NLST data set: 0.28) were also significant (all *P* < .001). Calibration plots are provided in eFigure 2 in the Supplement. The PLCO calibration slope was 1.17, indicating slight underestimation of observed 12-year mortality. The NLST calibration slope was approximately halved at 0.55, as would be expected given that 12-year mortality was predicted while 6-year mortality was observed. Deviation from the regression line was low, with an *R*^2^ of 0.99.

### Test-Retest Reliability

The CXR-risk test-retest reliability based on 2 different radiographs was assessed in 573 PLCO test participants whose T1 chest radiograph was repeated for quality control issues, with an intraclass correlation coefficient of 0.89 (95% CI, 0.88-0.91).

## Discussion

In this study, the deep learning CXR-risk score identified persons at low and high risk for long-term mortality based on a single chest radiograph. Persons with a very high CXR-risk score had a 53% mortality rate at 12 years in the PLCO data set and 34% at 6 years in the NLST data set, 18- and 15-fold higher compared with the very low-risk category. In both trials, prognostic value was complementary to the radiologists’ diagnostic findings (eg, lung nodule) and standard risk factors (eg, age, sex, and diabetes), with aHRs for death of 4.8 in the PLCO data set and 7.0 in the NLST data set. The CXR-risk score was also independently associated with lung cancer death (aHR, 11.1 and 8.4), as well as noncancer cardiovascular (aHR, 3.6 and 47.8) and respiratory (aHR, 27.5 and 31.9) death in both PLCO and NLST test data sets, respectively.

To our knowledge, this was the first report of deep learning to predict long-term prognosis from chest radiographs. The results extend observations based on other types of screening imaging. A deep learning model to predict 5-year major adverse cardiovascular events from fundoscopic eye images was developed in 48 101 UK Biobank healthy volunteers.^32^ As tested in 11 835 UK Biobank participants, the model predicted major adverse cardiovascular events but was not incremental to risk factors. A second deep learning model to predict 3-year all-cause mortality from chest computed tomography was developed in 7983 smokers in the COPDGene study.^33^ When tested in 1000 COPDGene participants and 1672 Evaluation of COPD Longitudinally to Identify Predictive Surrogate End Points (ECLIPSE) participants, the unadjusted HR ranged from 1.6 to 2.7. Taken as a whole, these and our data suggest that deep learning can extract prognostic information from existing diagnostic imaging.

Prognostic value was independent of radiographic findings traditionally used to diagnose lung cancer, such as lung nodules and lymphadenopathy. The CXR-risk score predicted multiple causes of death, including both lung cancer and noncancer death due to cardiovascular and respiratory illness. In fact, most deaths were from causes other than lung cancer (eTable 4 in the Supplement). These observations suggest that this CNN should not be considered as a lung cancer detector. Instead, we speculate that it identified patterns on the chest radiograph not tied to a single diagnosis or disease but as a summary measure of underlying prognosis and health. This concept of shared risk factors has been established for other biomarkers.^34^ For example, traditional cardiovascular risk factors, the coronary artery calcium score, and anti-inflammatory interleukin-1β therapy are associated with both cardiovascular disease and incident cancer.^35,36,37^

The CXR-risk CNN was tested in data sets from the PLCO and NLST, 2 independent, well-curated, multicenter randomized clinical trials of lung cancer screening in the community. The PLCO followed up nonsmokers and smokers for a median of 12 years; NLST included a heavy smoking population with median 6-year follow-up. Despite these differences, the CXR-risk score stratified persons into risk categories with a similar number of deaths per 1000 person-years (Table 2), suggesting generalizability. There was substantial improvement in AUC vs the radiologists’ chest radiograph findings. Improvement in AUC vs risk factors was modest but similar to that reported for adding the coronary artery calcium score, a guidelines-supported prognostic imaging marker,^38^ to risk factors in the Multi-Ethnic Study of Atherosclerosis (AUC of 0.79 to 0.83 for 4-year major coronary events).^39^

The trained model takes less than half a second to render a prediction from an existing chest radiograph. How could these predictions be used in practice?^40^ Like other risk scores for all-cause mortality,^7^ the CXR-risk score provides a summary measure of health and longevity but does not specify a disease to be treated. Nevertheless, there was an independent association with lung cancer death, even within the NLST cohort of long-term heavy smokers who would be conventionally considered to be at high risk. Similar associations with noncancer cardiovascular and respiratory death were seen in both data sets. For persons in the high- and very high-risk categories, a reasonable first step would be to confirm guidelines-appropriate lung cancer screening with computed tomography, as well as cardiovascular and respiratory primary prevention.^41,42,43^ This is important because currently 95% of lung cancer screening–eligible persons do not have screening computed tomography,^18,44^ and statin therapy is not taken by one-third of persons for whom it is recommended.^45^ Future iterations of the CXR-risk score could be fine-tuned for specific disease outcomes (eg, myocardial infarction) to complement existing risk factors and scores.^38^ The clinical effect is yet to be defined but conceivably could help inform decisions about lifestyle, screening, and prevention. On a population level, identifying those at greatest risk could help health systems allocate resources. From a research standpoint, the CXR-risk score could be used for trial cohort enrichment or risk adjustment. The potential for unintended harms, including unnecessary testing, denial of treatment, denial of insurance, worsening health disparities, and anxiety, should also be considered. As with polygenic risk scores, there is the potential to provide prognosis without the promise of a treatment to improve risk.^46^ Prospective clinical trials are needed to assess the effect on decision making and health outcomes.^47^

**Figure 3.**
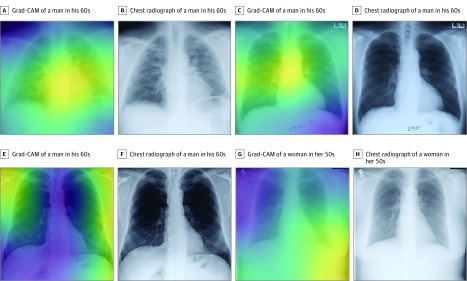
Gradient-Weighted Class Activation Maps (Grad-CAM) of Anatomy Contributing to the CXR-Risk Score A and B, Grad-CAM (A) and chest radiograph (B) of a man in his 60s from the Prostate, Lung, Colorectal, and Ovarian (PLCO) trial who died of respiratory illness in 2 years. Grad-CAM highlights an enlarged heart with prominent pulmonary vasculature indicating pulmonary edema (very high-risk CXR-risk score). C and D, Grad-CAM (C) and chest radiograph (D) of a man in his 60s in the PLCO trial who died of cardiovascular illness in 7 years. Grad-CAM highlights the mediastinum and aortic knob, which may indicate cardiovascular health; sternotomy wires indicate previous cardiothoracic surgery (very high-risk CXR-risk score). E and F, Grad-CAM (E) and chest radiograph (F) of a man in his 60s in the National Lung Screening Trial who was alive at the end of 6-years follow-up. Grad-CAM highlights the extrathoracic soft-tissues, which may reflect body habitus (low-risk CXR-risk score). G and H, Grad-CAM (G) and chest radiograph (H) of a woman in her 50s in the PLCO trial who was alive at the end of 9-years follow-up. Grad-CAM highlights the shadow of the left breast and waist, which convey information about sex and habitus, important determinants of longevity (very low-risk CXR-risk score).

Based on these potential implications, it will be important to understand the basis for individual predictions. Class activation maps (Figure 3) localize the anatomy contributing to the CXR-risk score. The cardiomediastinal silhouette, including the aortic knob and heart, were common focal points and consistent with the observed predictive power for cardiovascular and respiratory death. Activations in the lower contour of the breasts and chest wall impart information about age, sex, and habitus, all of which are important factors for longevity. Class activation maps should be interpreted with caution; whereas they localize anatomic features used to make predictions, what about that anatomy led to the prediction is open to interpretation. Ongoing work toward explaining individual predictions will be crucial for physician and patient acceptance of prognostic CNNs.^48^

The CXR-risk score took as input the radiograph only. This was intended to prove a point—that a CNN can extract prognostic information embedded in the image, without any other demographic or clinical information. Future deep learning models that incorporate this additional information, including age, sex, other risk factors, blood biomarkers, other imaging and nonimaging tests, and change over time will likely have greater prognostic value. Accuracy may also be further improved by training the CNN against survival with knowledge of the time to event and censoring,^49,50,51^ increasing the image resolution to allow detection of subtle abnormalities^52^ and with emerging CNN architectures.

### Limitations

Our analysis has limitations. The CNN was developed and tested in asymptomatic persons aged 55 to 74 years who had screening posterior-anterior chest radiographs. Whether these findings generalize to symptomatic populations and to other radiographic techniques is unknown. Most PLCO (87%) and NLST (93%) participants were of non-Hispanic white race/ethnicity; prognostic value will need to be evaluated among other demographic groups.^53^

## Conclusions

The results suggest that the CXR-risk CNN can stratify the risk of long-term mortality using chest radiographs. Individuals at high risk may benefit from prevention, screening, and lifestyle interventions. Further research is necessary to determine how this can improve individual and population health.

## Notes

Supplement. **eTable 1.** Risk Thresholds for the CXR-Risk Score **eTable 2.** CXR-Risk Score Hazard Ratios for All-Cause Mortality, Unadjusted and Adjusted for Radiograph Findings, Risk Factors, and the Combination of Findings Plus Risk Factors **eTable 3.** Cox Model Including the CXR-Risk Score, Risk Factors, and Radiograph Findings With Adjusted Hazard Ratios for All-Cause Mortality **eTable 4.** Cause-Specific Mortality by CXR-Risk Score **eTable 5.** Area Under the Receiver Operating Characteristic Curve (AUC) and Continuous Net Reclassification Index (NRI) for All-Cause Mortality **eFigure 1.** CXR-Risk Score and 12-Year Mortality, Stratified by Sex and Age **eFigure 2.** CXR-Risk Calibration Plots **eMethods.** Determination of Cause of Death; Model Development; Chest Radiograph Image Processing and Data Augmentation; Classifier, Architecture and Training; Implementation **eReferences.**
