# Supplementary material for: Deep Learning to Assess Long-term Mortality From Chest Radiographs
Source: JAMA Netw Open. 2019 Jul 19;2(7):e197416. doi: 10.1001/jamanetworkopen.2019.7416 (PMC6646994; doi:10.1001/jamanetworkopen.2019.7416)
Supplement: Supplement. — eTable 1. Risk Thresholds for the CXR-Risk Score eTable 2. CXR-Risk Score Hazard Ratios for All-Cause Mortality, Unadjusted and Adjusted for Radiograph Findings, Risk Factors, and the Combination of Findings Plus Risk Factors eTable 3. Cox Model Including the CXR-Risk Score, Risk Factors, and Radiograph Findings With Adjusted Hazard Ratios for All-Cause Mortality eTable 4. Cause-Specific Mortality by CXR-Risk Score eTable 5. Area Under the Receiver Operating Characteristic Curve (AUC) and Continuous Net Reclassification Index (NRI) for All-Cause Mortality eFigure 1. CXR-Risk Score and 12-Year Mortality, Stratified by Sex and Age eFigure 2. CXR-Risk Calibration Plots eMethods. Determination of Cause of Death; Model Development; Chest Radiograph Image Processing and Data Augmentation; Classifier, Architecture and Training; Implementation eReferences. [file jamanetwopen-2-e197416-s001.pdf]

Supplementary Online Content

Lu MT, Ivanov A, Mayrhofer T, Hosny A, Aerts HJWL, Hoffmann U. Deep learning to assess long-term mortality from chest radiographs. *JAMA Netw Open*. 2019;2(7):e197416. doi:10.1001/jamanetworkopen.2019.7416

eTable 1. Risk Thresholds for the CXR-Risk Score

eTable 2. CXR-Risk Score Hazard Ratios for All-Cause Mortality, Unadjusted and Adjusted for Radiograph Findings, Risk Factors, and the Combination of Findings Plus Risk Factors

eTable 3. Cox Model Including the CXR-Risk Score, Risk Factors, and Radiograph Findings With Adjusted Hazard Ratios for All-Cause Mortality

eTable 4. Cause-Specific Mortality by CXR-Risk Score

eTable 5. Area Under the Receiver Operating Characteristic Curve (AUC) and Continuous Net Reclassification Index (NRI) for All-Cause Mortality

eFigure 1. CXR-Risk Score and 12-Year Mortality, Stratified by Sex and Age

eFigure 2. CXR-Risk Calibration Plots

eMethods. Determination of Cause of Death; Model Development; Chest Radiograph Image Processing and Data Augmentation; Classifier, Architecture and Training; Implementation

eReferences

This supplementary material has been provided by the authors to give readers additional information about their work.

© 2019 Lu MT et al. *JAMA Network Open.*

eTable 1. Risk Thresholds for the CXR-Risk Score.

Risk thresholds defining the CXR-risk score and percentage falling within each category. Thresholds were set in the PLCO development data set based on quantiles, then applied to the PLCO and NLST test data sets.

CXR-risk score	CNN probability threshold	PLCO development (n=41,856)	PLCO test (n=10,464)	NLST test (n=5,493)
**Very low**	0	25.0% (10,465)	24.3% (2,543)	13.7% (752)
**Low**	0.063583	25.0% (10,464)	26.5% (2,769)	30.6% (1,679)
**Moderate**	0.096197	25.0% (10,463)	25.6% (2,674)	31.4% (1,723)
**High**	0.1579735	20.0% (8,372)	19.2% (2,006)	21.1% (1,159)
**Very high**	0.34843	5.0% (2,092)	4.5% (472)	3.3% (180)

© 2019 Lu MT et al. *JAMA Network Open.*

eTable 2. CXR-Risk Score Hazard Ratios for All-Cause Mortality, Unadjusted and Adjusted for Radiograph Findings, Risk Factors, and the Combination of Findings Plus Risk Factors.

**PLCO test data set**								
**CXR-risk score**	**Unadjusted HR (95% CI)**	**P**	**HR adjusted for CXR findings (95% CI)**	**P**	**HR adjusted for risk factors (95% CI)**	**P**	**HR adjusted for findings & risk factors (95% CI)**	**P**
Very low	1 [Reference]		1 [Reference]		1 [Reference]		1 [Reference]	
Low	2.0 (1.6-2.6)	<0.001	2.0 (1.6-2.5)	<0.001	1.5 (1.1-1.9)	0.002	1.4 (1.1-1.8)	0.003
Moderate	3.3 (2.7-4.2)	<0.001	3.2 (2.6-4.0)	<0.001	1.8 (1.4-2.2)	<0.001	1.7 (1.3-2.2)	<0.001
High	7.0 (5.6-8.6)	<0.001	6.5 (5.2-8.1)	<0.001	2.8 (2.2-3.6)	<0.001	2.6 (2.1-3.4)	<0.001
Very high	18.3 (14.5-23.2)	<0.001	16.1 (12.6-20.5)	<0.001	5.4 (4.1-7.1)	<0.001	4.8 (3.6-6.4)	<0.001
**NLST test data set**								
**CXR-risk score**	**Unadjusted HR (95% CI)**	**P**	**HR adjusted for CXR findings (95% CI)**	**P**	**HR adjusted for risk factors (95% CI)**	**P**	**HR adjusted for findings & risk factors (95% CI)**	**P**
Very low	1 [Reference]		1 [Reference]		1 [Reference]		1 [Reference]	
Low	1.4 (0.87-2.4)	0.158	1.4 (0.86-2.3)	0.17	1.2 (0.81-2.0)	0.53	1.2 (0.70-1.9)	0.56
Moderate	2.6 (1.6-4.1)	<0.001	2.5 (1.5-4.0)	<0.001	1.8 (1.1-2.9)	0.020	1.7 (1.0-2.8)	0.034
High	3.9 (2.4-6.3)	<0.001	3.5 (2.2-5.7)	<0.001	2.5 (1.5-4.1)	<0.001	2.3 (1.4-3.7)	0.002
Very high	15.2 (9.2-25.3)	<0.001	12.6 (7.5-21.1)	<0.001	8.3 (4.8-14.2)	<0.001	7.0 (4.0-12.1)	<0.001

CXR = chest radiograph, HR = hazard ratio, CI = confidence interval
Adjusted hazard ratios are adjusted for 9 chest radiograph findings (lung nodule, major atelectasis, pleural plaque or effusion, lymphadenopathy, chest wall or bony lesion, COPD/emphysema, cardiomegaly or other cardiovascular abnormality, and lung fibrosis), 10 risk factors (age, sex, smoking category, diabetes, hypertension, obesity, underweight, past myocardial infarction, past stroke, and past cancer), or the combination of findings plus risk factors.

© 2019 Lu MT et al. *JAMA Network Open.*

eTable 3. Cox Model Including the CXR-Risk Score, Risk Factors, and Radiograph Findings With Adjusted Hazard Ratios for All-Cause Mortality

		PLCO Test (n=10,464)		NLST Test (n=5,493)	
		aHR (95% CI)	P	aHR (95% CI)	p
**CXR-risk score**	Very low	1 [Reference]		1 [Reference]	
	Low	1.4 (1.1-1.8)	0.003	1.2 (0.70-1.9)	0.56
	Moderate	1.7 (1.3-2.2)	<0.001	1.7 (1.0-2.8)	0.034
	High	2.6 (2.1-3.4)	<0.001	2.3 (1.4-3.7)	0.002
	Very high	4.8 (3.6-6.4)	<0.001	7.0 (4.0-12.1)	<0.001
**Risk factors**	Age (years)	1.1 (1.1-1.1)	<0.001	1.0 (1.0-1.1)	<0.001
	Male sex	1.5 (1.3-1.7)	<0.001	1.2 (0.93-1.5)	0.19
	Smoking<br>Never<br>Former<br>Current	 1 [Reference]<br>1.3 (1.2-1.5)<br>2.6 (2.2-3.1)	 <br><0.001<br><0.001	 NA<br>1 [Reference]<br>1.6 (1.3-2.0)	 <br><br><0.001
	Diabetes	1.7 (1.5-2.0)	<0.001	1.5 (1.1-2.0)	0.016
	Hypertension	1.3 (1.2-1.5)	<0.001	1.4 (1.1-1.7)	0.004
	Obesity (BMI ≥30kg/m2)	1.1 (0.98-1.3)	0.12	1.0 (0.82-1.3)	0.71
	Underweight (BMI <18.5 kg/m2)	1.4 (0.89-2.3)	0.15	3.6 (1.9-6.8)	<0.001
	Past myocardial infarction	1.3 (1.2-1.5)	<0.001	1.3 (1.0-1.8)	0.032
	Past stroke	1.8 (1.5-2.3)	<0.001	1.3 (0.84-2.0)	0.24
	Past cancer	1.5 (1.2-1.9)	<0.001	1.1 (0.68-1.9)	0.65
**Radiograph findings**	Lung nodule	1.3 (1.1-1.5)	0.006	1.6 (1.2-2.1)	0.001
	Major atelectasis	1.8 (0.56-5.6)	0.33	1.2 (0.29-4.9)	0.81
	Pleural plaque or effusion	0.88 (0.69-1.1)	0.30	1.1 (0.68-1.7)	0.78
	Lymphadenopathy	1.0 (0.59-1.9)	0.87	3.0 (0.95-9.5)	0.060
	Chest wall or bony abnormality	1.1 (0.83-1.4)	0.65	1.8 (0.64-4.8)	0.28
	Lung opacity	1.4 (0.87-2.2)	0.16	3.6 (1.2-10.4)	0.018
	COPD/emphysema	1.4 (1.1-1.7)	0.014	1.1 (0.85-1.5)	0.43
	Cardiomegaly or other cardiovascular abnormality	1.4 (1.1-1.7)	0.004	1.8 (1.0-3.1)	0.045
	Lung fibrosis	1.2 (1.0-1.4)	0.021	1.3 (0.94-1.9)	0.10

BMI = body mass index, COPD = chronic obstructive pulmonary disease, aHR = adjusted hazard ratio, CI = confidence interval, NA = not applicable
Adjusted hazard ratios are adjusted for chest radiograph findings (lung nodule, major atelectasis, pleural plaque or effusion, lymphadenopathy, chest wall or bony lesion, COPD/emphysema, cardiomegaly or other cardiovascular abnormality, and lung fibrosis) and risk factors (age, sex, smoking category, diabetes, hypertension, obesity, underweight, past myocardial infarction, past stroke, and past cancer).

© 2019 Lu MT et al. *JAMA Network Open.*

eTable 4. Cause-Specific Mortality by CXR-Risk Score

The CXR-risk score predicted multiple causes of death, including lung cancer death, cardiovascular death, and respiratory death. This association was robust to adjustment for the radiologists' findings (e.g. lung nodule) and standard risk factors (e.g. age, sex, diabetes).

A) PLCO test data set

Cause of death	CXR-risk score	Mortality	Unadjusted HR (95% CI)	P	Adjusted HR (95% CI)	P
**Lung cancer**	Very low	0.28% (7/2,543)	REF		REF	
	Low	0.61% (17/2,769)	2.2 (0.9-5.3)	0.078	1.8 (0.7-4.3)	0.198
	Moderate	1.4% (37/2,674)	5.1 (2.3-11.4)	<0.001	3.1 (1.3-7.2)	0.008
	High	3.2% (64/2,006)	12.5 (5.7-17.2)	<0.001	5.7 (2.4-13.1)	<0.001
	Very high	7.2% (34/472)	34.8 (15.4-78.5)	<0.001	11.1 (4.4-27.8)	<0.001
	All	1.5% (159/10,464)				
**Non-lung cancer**	Very low	1.7% (43/2,543)	REF		REF	
	Low	3.0% (82/2,769)	1.7 (1.2-2.5)	0.004	1.2 (0.8-1.8)	0.28
	Moderate	3.7% (99/2,674)	2.2 (1.5-3.1)	<0.001	1.1 (0.8-1.7)	0.48
	High	5.9% (118/2,006)	3.7 (2.6-5.3)	<0.001	1.6 (1.0-2.4)	0.029
	Very high	9.7% (46/472)	7.6 (5.0-11.6)	<0.001	2.5 (1.5-4.2)	<0.001
	All	3.7% (388/10,464)				
**Cardiovascular illness**	Very low	0.90% (23/2,543)	REF		REF	
	Low	2.3% (64/2,769)	2.5 (1.6-4.1)	<0.001	1.6 (1.0-2.5)	0.067
	Moderate	4.0% (107/2,674)	4.4 (2.8-7.0)	<0.001	1.8 (1.1-2.9)	0.018
	High	7.9% (159/2,006)	9.3 (6.0-14.4)	<0.001	2.4 (1.4-3.8)	0.001
	Very high	16.7% (79/472)	24.1 (15.2-38.4)	<0.001	3.6 (2.1-6.2)	<0.001
	All	4.1% (432/10,464)				
**Respiratory illness**	Very low	0.12% (3/2,543)	REF		REF	
	Low	0.47% (13/2,769)	3.9 (1.1-13.6)	0.034	2.9 (0.8-10.3)	0.096

© 2019 Lu MT et al. *JAMA Network Open.*

	Moderate	0.79% (21/2,674)	6.6 (2.0-22.1)	0.002	3.5 (1.0-12.0)	0.051
	High	2.4% (48/2,006)	21.4 (6.7-68.7)	<0.001	8.3 (2.4-28.1)	0.001
	Very high	9.5% (45/472)	107.8 (33.5-347.0)	<0.001	27.5 (7.7-97.8)	<0.001
	All	1.2% (130/10,464)				
**Other***	Very low	0.83% (21/2,543)	REF		REF	
	Low	1.4% (40/2,769)	1.7 (1.0-2.9)	0.047	1.3 (0.8-2.3)	0.28
	Moderate	2.8% (75/2,674)	3.4 (2.1-5.5)	<0.001	2.1 (1.2-3.5)	0.006
	High	5.5% (111/2,006)	7.1 (4.4-11.3)	<0.001	3.5 (2.1-6.0)	<0.001
	Very high	9.7% (46/472)	15.6 (9.3-26.1)	<0.001	6.2 (3.3-11.5)	<0.001
	All	2.8% (293/10,464)				

B) NLST test data set

Cause of death	CXR-risk score	Mortality	Unadjusted HR (95% CI)	P	Adjusted HR (95% CI)	P
**Lung cancer**	Very low	0.55% (4/732)	REF		REF	
	Low	1.7% (29/1,679)	3.2 (1.1-9.2)	0.027	3.0 (1.0-8.5)	0.041
	Moderate	2.0% (35/1,723)	3.9 (1.4-11.0)	0.010	3.1 (1.1-8.8)	0.039
	High	2.8% (33/1,159)	5.6 (2.0-15.9)	0.001	4.0 (1.4-11.8)	0.012
	Very high	6.7% (12/180)	14.9 (4.8-46.3)	<0.001	8.4 (2.5-28.0)	0.001
	All	2.1% (113/5,493)				
**Non-lung cancer**	Very low	1.6% (12/732)	REF		REF	
	Low	0.71% (12/1,679)	0.4 (0.2-1.0)	0.50	0.3 (0.-0.7)	0.008
	Moderate	0.99% (17/1,723)	0.6 (0.3-1.3)	0.23	0.4 (0.2-0.8)	0.017
	High	2.0% (23/1,159)	1.3 (0.7-2.7)	0.43	0.7 (0.3-1.6)	0.41
	Very high	1.7% (3/180)	1.3 (0.4-4.5)	0.70	0.6 (0.2-2.4)	0.50
	All	1.2% (67/5,493)				
**Cardiovascular illness**	Very low	0.14% (1/732)	REF		REF	
	Low	0.83% (14/1,679)	6.3 (0.8-47.8)	0.076	4.9 (0.6-37.7)	0.13
	Moderate	1.3% (23/1,723)	10.3 (1.4-76.2)	0.022	6.5 (0.9-49.1)	0.069
	High	2.4% (28/1,159)	19.1 (2.6-140.5)	0.004	11.6 (1.5-87.7)	0.018
	Very high	10.0% (18/180)	89.7 (12.0-672.0)	<0.001	47.8 (6.1-374.9)	<0.001

© 2019 Lu MT et al. *JAMA Network Open.*

	All	1.5% (84/5,493)				
**Respiratory illness**	Very low	0.14% (1/732)	REF		REF	
	Low	0.06% (1/1,679)	0.4 (0.03-7.2)	0.57	0.4 (0.02-5.6)	0.46
	Moderate	0.81% (14/1,723)	6.3 (0.8-47.6)	0.076	4.0 (0.5-31.6)	0.19
	High	1.1% (13/1,159)	8.8 (1.2-67.5)	0.036	4.6 (0.6-37.2)	0.16
	Very high	8.3% (15/180)	73.8 (9.7-558.5)	<0.001	31.9 (3.9-263.5)	0.001
	All	0.8% (44/5,493)				
**Other***	Very low	0.27% (2/732)	REF		REF	
	Low	0.48% (8/1,679)	1.8 (0.4-8.5)	0.46	1.4 (0.3-6.5)	0.70
	Moderate	1.5% (26/1,723)	5.8 (1.4-24.6)	0.016	3.2 (0.7-13.9)	0.12
	High	1.5% (17/1,159)	5.8 (1.4-25.2)	0.018	2.4 (0.5-10.9)	0.27
	Very high	7.2% (13/180)	32.4 (7.3-143.6)	<0.001	8.1 (1.6-)	0.010
	All	1.1% (66/5,493)				

HR = hazard ratio, CI = confidence interval
Adjusted hazard ratios are adjusted for 9 chest radiograph findings (lung nodule, major atelectasis, pleural plaque or effusion, lymphadenopathy, chest wall or bony lesion, COPD/emphysema, cardiomegaly or other cardiovascular abnormality, and lung fibrosis) and 10 risk factors (age, sex, smoking category, diabetes, hypertension, obesity, underweight, past myocardial infarction, past stroke, and past cancer).
*One subject with a missing death certificate is graded as “Other” cause of death in both the PLCO and NLST data sets

© 2019 Lu MT et al. *JAMA Network Open.*

eTable 5. Area Under the Receiver Operating Characteristic Curve (AUC) and Continuous Net Reclassification Index (NRI) for All-Cause Mortality

	PLCO Test (n=10,464, 12-year follow-up)					NLST Test (n=5,493, 6-year follow-up)				
	**AUC alone (95% CI)**	**AUC with CXR-risk (95% CI)**	**P for ΔAUC**	**NRI (95% CI)**	**P for NRI**	**AUC alone (95% CI)**	**AUC with CXR-risk (95% CI)**	**P for ΔAUC**	**NRI (95% CI)**	**P for NRI**
CXR-risk	0.75 (0.73-0.76)	NA	NA	NA	NA	0.68 (0.65-0.71)	NA	NA	NA	NA
Radiograph findings	0.58 (0.57-0.59)	0.74 (0.73-0.76)	<0.001	0.59 (0.53-0.65)	<0.001	0.59 (0.56-0.62)	0.70 (0.67-0.73)	<0.001	0.44 (0.33-0.55)	<0.001
Clinical risk factors	0.76 (0.75-0.78)	0.78 (0.77-0.79)	<0.001	0.21 (0.15-0.28)	<0.001	0.68 (0.65-0.71)	0.72 (0.69-0.75)	<0.001	0.32 (0.20-0.43)	<0.001
Risk factors + findings	0.76 (0.75-0.78)	0.78 (0.77-0.79)	<0.001	0.20 (0.13-0.27)	<0.001	0.70 (0.67-0.73)	0.73 (0.70-0.76)	<0.001	0.28 (0.17-0.41)	<0.001

Chest radiograph (CXR) findings include lung nodule, major atelectasis, pleural plaque or effusion, lymphadenopathy, chest wall or bony lesion, COPD/emphysema, cardiomegaly or other cardiovascular abnormality, and lung fibrosis. Risk factors include age, sex, smoking category, diabetes, hypertension, obesity, underweight, past myocardial infarction, past stroke, and past cancer.

© 2019 Lu MT et al. *JAMA Network Open.*

eFigure 1. CXR-Risk Score and 12-Year Mortality, Stratified by Sex and Age

CXR-risk score and 12-year mortality rate in the PLCO test data set, stratified by A) male and B) female sex and age in years

A) Men

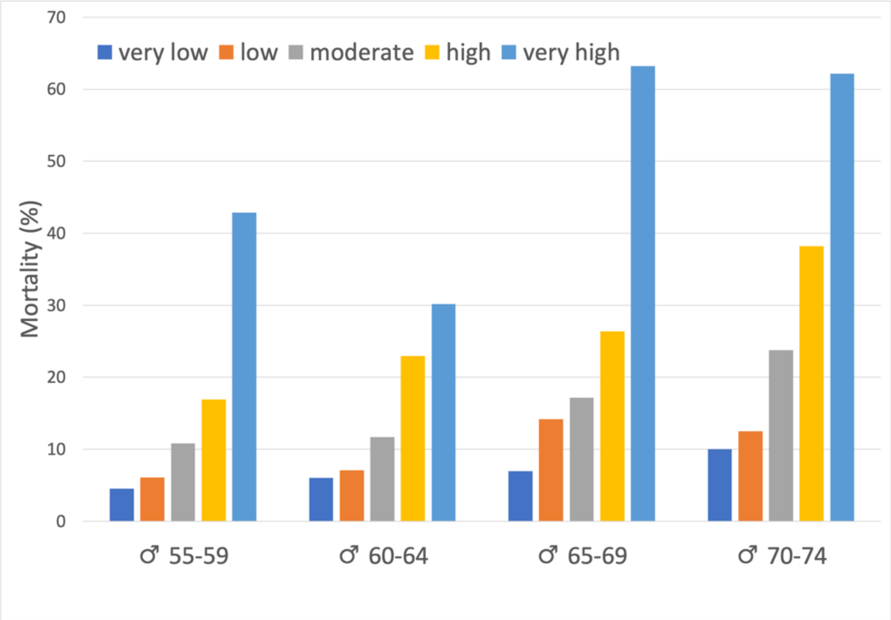

B) Women

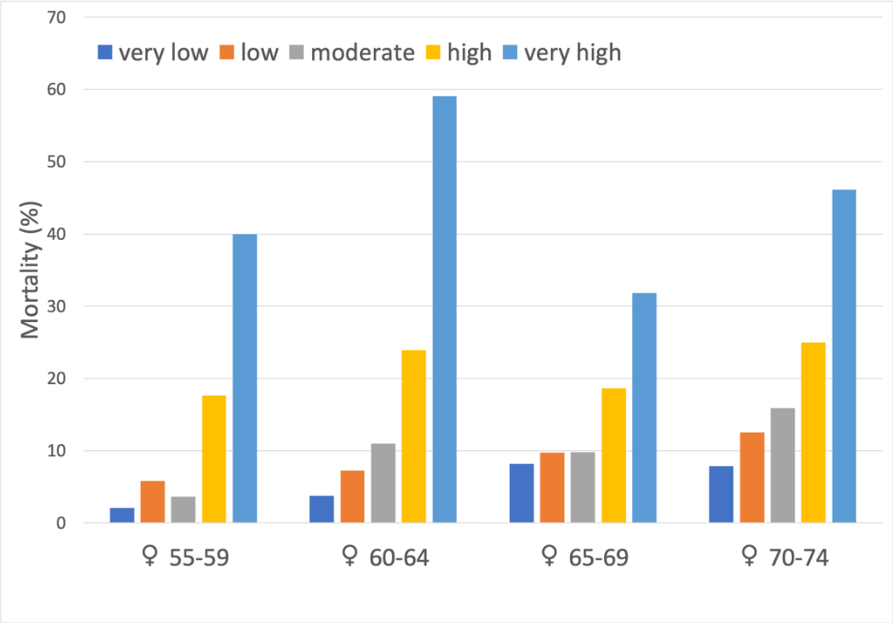

© 2019 Lu MT et al. *JAMA Network Open.*

eFigure 2. CXR-Risk Calibration Plots

Calibration plots of CXR-risk predicted 12-year mortality versus observed 12-year (PLCO test data set) and 6-year (NLST test data set) mortality.

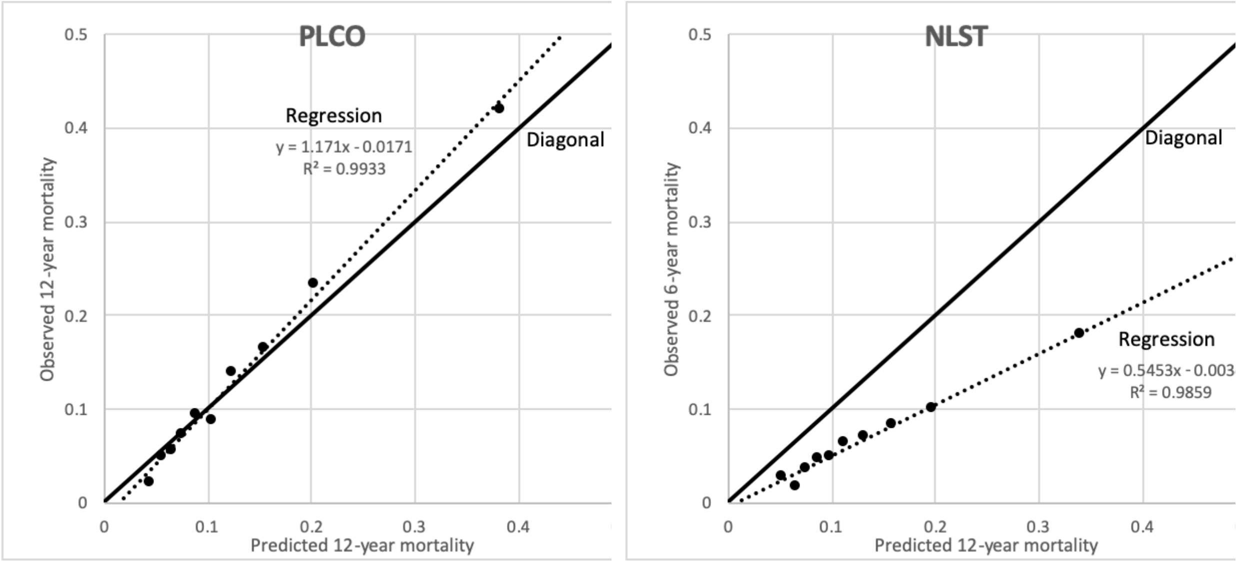

© 2019 Lu MT et al. *JAMA Network Open.*

eMethods. Determination of Cause of Death; Model Development; Chest Radiograph Image Processing and Data Augmentation; Classifier, Architecture and Training; Implementation

Determination of cause of death

Cause of death was reported according to the guidelines of the parent PLCO and NLST trials.[1,2] In PLCO, deaths possibly related to a PLCO cancer were reviewed by an independent adjudication committee who had access to death certificates, medical records, and autopsy reports.[1] Other causes of death were assigned based on review of death certificates.[3,4] In NLST, incident lung cancer was likewise reviewed by an independent adjudication committee. Cause of death was based on the death certificate's International Classification of Diseases, 10th Revision (ICD-10) code as defined in the parent trial publication: lung cancer (C33-C34), non-lung cancer (C00-D48 excluding C33-C34), cardiovascular illness (I00-I99), respiratory illness (J00-J99), and other.[2]

Model development

Chest radiograph image processing and data augmentation

PLCO chest radiographs were scanned at sites and collected by the NCI as tagged image format (.tif) files, with black bars redacting protected health information (PHI). The NLST chest radiographs available for our study were collected by ACRIN as anonymized Digital Imaging and Communications in Medicine (DICOM) files, with no black bars. PLCO and NLST chest radiographs were converted to portable network

© 2019 Lu MT et al. *JAMA Network Open.*

graphics (.png) format. For the PLCO development (training and tuning) data sets, each participant's baseline (T0) and year 1 (T1) chest radiographs were included and treated independently. For the PLCO test and NSLT test data sets, only the first baseline chest radiograph was included, to reflect the anticipated clinical use case.

For regularization, geometric data augmentation of the images included random rotation up to ±2.5 degrees, brightness ±15%, and zoom factor up to 20%. The image was then resized to the target resolution along its lesser dimension and randomly cropped to a square.[5] No flipping augmentation was performed, to preserve the anatomic situs of the heart and mediastinum. Images were progressively resized during model development, starting with 128x128 pixel images with a batch size of 512 and ending with 224x224 pixel images with a batch size of 368.[2] The mixup data augmentation principle, where the model was trained using combinations of images and their labels, was employed for the training images only with an alpha of 0.4.[6] Test time augmentation was performed, with a reduced scaling factor of 5% followed by a resize and random square crop to 224x224 pixels including each of the four corners of the image.[7]

Classifier, architecture and training

The goal was to predict all-cause mortality. However only 13% of PLCO participants died. Due to this unbalanced distribution of the desired classifier (death), we chose to train with a staged classifier. In the first stage of training, the CNN was trained against a composite binary classifier of death or incident cancer, which occurred in 25% of PLCO development data set participants. In epidemiology, a composite outcome including

© 2019 Lu MT et al. *JAMA Network Open.*

both nonfatal endpoints (e.g. incident cancer) and death is commonly used when the primary outcome (death) is rare.[8] The rationale is that these nonfatal outcomes are along the causal pathway towards death and can be considered as a surrogate endpoint. We extend this intuition to the classifier, under the assumption that persons who develop cancer share similar underlying risk to those who died. In the second stage of training, the classifier was narrowed to death. Results from the final model are reported for death only. The model was developed using the chest radiograph images and the classifier only—no other information, including age, sex, risk factors, radiographic findings, duration of follow-up, or censoring was available to the CNN.

A PyTorch implementation[9] of the Inception v4 architecture[10] was modified by replacing the final "head" of average pooling and linear layers with a "custom head" of adaptive concatenation pooling, batch normalization, dropout, linear, ReLU, batch normalization, dropout and a final linear layer.[7] The "body" of the network was initialized with pre-trained weights from the 2015 ImageNet classification challenge as a starting point.[9,11] Mixed precision training[12] using the ADAM optimizer[13] was employed, with the one cycle policy to govern learning rate and momentum.[14] Differential learning rates were used, with a maximum learning rate of up to 1e-02 in the head and lesser learning rates in the body.[7,15-17]

Implementation

The CXR-risk model was developed using open source and/or freely available software with off-the-shelf consumer-grade hardware. Software included PyTorch 1.0, Python

© 2019 Lu MT et al. *JAMA Network Open.*

3.7, and the fastai deep learning libraries.[7] The final CNN was developed using a Nvidia Titan RTX graphics processing unit (GPU) with 24 GB of video RAM on an AMD 1950x platform with Ubuntu Linux 16.04. Image pre-processing was performed with ImageMagick (ImageMagick Studio, Landenberg PA) and DCMTK (OFFIS, Oldenburg Germany).

© 2019 Lu MT et al. *JAMA Network Open.*

eReferences

1. Oken MM, Hocking WG, Kvale PA, et al. Screening by chest radiograph and lung cancer mortality: the Prostate, Lung, Colorectal, and Ovarian (PLCO) randomized trial. *JAMA.* 2011;306(17):1865-1873.
2. NLST Research Team, Aberle DR, Adams AM, et al. Reduced lung-cancer mortality with low-dose computed tomographic screening. *N Engl J Med.* 2011;365(5):395-409.
3. Prorok PC, Andriole GL, Bresalier RS, et al. Design of the Prostate, Lung, Colorectal and Ovarian (PLCO) Cancer Screening Trial. *Control Clin Trials.* 2000;21(6 Suppl):273S-309S.
4. Miller AB, Yurgalevitch S, Weissfeld JL, Prostate LC, Ovarian Cancer Screening Trial Project T. Death review process in the Prostate, Lung, Colorectal and Ovarian (PLCO) Cancer Screening Trial. *Control Clin Trials.* 2000;21(6 Suppl):400S-406S.
5. Howard AG. Some Improvements on Deep Convolutional Neural Network Based Image Classification. *ArXiv e-prints* 2013;arXiv:1312.5402. https://arxiv.org/abs/1312.5402. Accessed June 01, 2018.
6. Zhang H, Cisse M, Dauphin YN, Lopez-Paz D. Mixup: Beyond empirical risk minimization. *ArXiv e-prints 2017; h*ttps://arxiv.org/abs/1710.09412*.* Accessed November 12, 2018.
7. Howard J. Fastai libraries. 2019; https://github.com/fastai/fastai. Accessed January 24, 2019.

© 2019 Lu MT et al. *JAMA Network Open.*

8. Freemantle N, Calvert M, Wood J, Eastaugh J, Griffin C. Composite outcomes in randomized trials: greater precision but with greater uncertainty? *JAMA.* 2003;289(19):2554-2559.

9. Cadene R. Pretrained models for Pytorch. 2019; https://github.com/Cadene/pretrained-models.pytorch. Accessed January 3, 2019.

10. Szegedy C, Ioffe S, Vanhoucke V, Alemi A. *Inception-v4, Inception-ResNet and the Impact of Residual Connections on Learning. ArXiv e-prints* 2016. https://arxiv.org/abs/1602.07261. Accessed November 12, 2018.

11. Russakovsky O, Deng J, Su H, et al. ImageNet Large Scale Visual Recognition Challenge. *International Journal of Computer Vision.* 2015;115(3):211-252.

12. Micikevicius P, Narang S, Alben J, et al. Mixed Precision Training. *ArXiv e-prints.* 2017. https://arxiv.org/abs/1710.03740. Accessed August 12, 2018.

13. Kingma DP, Ba J. Adam: A Method for Stochastic Optimization. *ArXiv e-prints.* 2014. https://arxiv.org/abs/1412.6980. Accessed February 12, 2018.

14. Smith LN. A disciplined approach to neural network hyper-parameters: Part 1 -- learning rate, batch size, momentum, and weight decay. *ArXiv e-prints.* 2018. https://arxiv.org/abs/1803.09820. Accessed July 3, 2018.

15. Smith LN. Cyclical Learning Rates for Training Neural Networks. *ArXiv e-prints.* 2015. https://ui.adsabs.harvard.edu/#abs/2015arXiv150601186S. Accessed June 01, 2015.

16. Huang G, Li Y, Pleiss G, Liu Z, Hopcroft JE, Weinberger KQ. Snapshot Ensembles: Train 1, get M for free. *ArXiv e-prints.* 2017.

© 2019 Lu MT et al. *JAMA Network Open.*

https://ui.adsabs.harvard.edu/#abs/2017arXiv170400109H. Accessed March 01, 2017.

17. Loshchilov I, Hutter F. SGDR: Stochastic Gradient Descent with Warm Restarts. *ArXiv e-prints.* 2016. https://ui.adsabs.harvard.edu/#abs/2016arXiv160803983L. Accessed August 01, 2017.
